# Etiology of Tubercular Disease

**Published:** 1885-09

**Authors:** J. W. McLaughlin

**Affiliations:** President Texas Microscopic Association, Austin, Texas


### ETIOLOGY OF TUBERCULAR DISEASES.

*By J. W. McLaughlin, M. D., President Texas Microscopic Association, Austin, Texas.*

For Daniel's Texas Medical Journal.

PERHAPS no subject, within the range of medicine, has excited more discussion, developed more conflicting opinions, or, until within a very recent time, has so successfully eluded the investigations of science, as that which heads this article.

The extensive prevalence of phthisis in all parts of the world, and its remarkable fatality, which (according to Hirsch) comprises two-sevenths of the deaths which occur from all causes, should impress every physician with the importance of keeping his knowledge of this disease abreast of that of his age.

Phthisis was clearly described by the physicians of antiquity. Hippocrates ascribed it to suppuration of the lungs and pleura. Sylvius recognized nodes in the lungs which, he thought, were enlarged pulmonary glands. Mangetus first compared these nodes to millet seed in size, and found them in the liver, spleen and kidneys of the phthisical, as well as in their lungs. Matthew Baille first differentiated these tubercles from glands,—he also taught that the softening and breaking down of these tubercles produced cavities. Bayle announced that phthisis was a distinctly specific or infectious disease, frequently associated with pulmonary inflammations, catarrhs, hæmoptysis, etc., but was never produced by them. Next came Laeneck's thesis concerning the gray granulations and their change into yellow tubercles. His teachings greatly simplified the whole subject; he recognized but one phthisis—a "phthisis tuberculosa"—and regarded tubercle, whether infiltrated or in isolated bodies, whether gray or yellow, as being the same substance. The views of Laeneck, through the teachings of Andral, Louis, Rokitansky, and others, became very popular. A few pathologists objected to them because they assumed that all cheesy masses found in the lungs, or other portions of the body, were the result of tuberculosis—in fact, were cheesy tubercles. These causes for disbelief in Laeneck's theory resulted in the doctrine promulgated by Virchow, that no process was to be called tubercular unless gray milliary tubercles were found. According to this theory, the most important form of this disease—viz: phthisis—is almost entirely removed from the domain of tuberculosis.

The next theory was announced by Buhl, that acute disseminated milliary tuberculosis was a "resorption disease," which always originated from a cheesy mass, usually a cheesy lymphatic gland, located in some portions of the body. The tubercles, he states, are found in greatest numbers around this focus, as though it were the point of inoculation, and the tubercles the result of resorption. In this way, milliary tuberculosis lost somewhat its character of a primary lesion, and seemed rather the result of resorption, and dependent upon some anterior condition.

The subject was now taken up by the experimental pathologists. Villeman and Klebs revived the old doctrine of Baille regarding the infectious nature of tuberculosis. They established this claim by inoculation experiments. In many hundreds of cases they transmitted tuberculosis to the lower animals by inoculating them with tuberculous matter.

These experiments have been confirmed by many others. Frankle and Conheim insist that the manner and matter of inoculation make no difference in certain animals. In fact, inoculation in the rabbit and guinea pig is unnecessary ; that the formation of a focus of suppuration is all that is required to render these animals tuberculous. This unexpected result of inoculation experiments relegated the whole subject to its original condition, where it remained until the discoveries of Koch have made us masters of the situation.

In connection with this subject, I must call your attention to an important fact regarding the behavior of phthisis. I refer to the similarity between the predisposition of certain animals for tuberculosis,and its almost exclusive occurrence in a certain group of persons—the scrofulous.

Inflammation in this constitutional disease runs a chronic course ; whilst its products disappear slowly,or remain stationary, and undergo degenerative changes of a cheesy nature, thereby furnishing a nidus for the growth of the tubercle bacilli.

I shall next invite your attention to the histology of tubercle. When seen under the microscope in its fresh state, a gray milliary tubercle is a little circular nodule composed of cell elements, derived mainly from the white blood cells, in part from the endothelium of the vessels and the connective tissue cells. These are held together by a net-work of fibrillar connective tissue. In the center of each tubercle or nodule we usually find one or more large multi-nuclear or "giant cells." Outside of these are seen smaller elements, round or fusiform in shape, known as "epithelioid cells," whilst near the periphery of the tubercle are seen small round or "lymphoid cells." A tubercle is essentially a non-vascular growth, as new formed vessels are not found in it. In consequence of this, its center soon becomes necrotic or caseous.

Tubercles may be deposited singly, in groups, or as infiltrations. In whatever form deposited, they possess the same life-history and undergo the degenerative changes above described.

Pathologists do not agree as regards the significance of the different cells found in tubercles. Some of them claim that the large "giant" and "epithelioid" cells are distinctive of tubercle, and speak of them as "tubercle cells"—in fact, base the diagnosis of tuberculosis upon their presence.

Unfortunately for this assumption, these giant and epithelioid cells are not confined to tubercles. They are formed in the early stages of reparative inflammations, and are the cells from which granulative tissue is formed, so that the distinguishing features of tubercle do not reside in its anatomical elements.

The life history of tubercle is what distinguishes it from other neoplasms, viz: a definite nodule of a certain size, which contains no new formed vessels, and which, at a certain stage of its growth ceases to progress, and undergoes necrotic or degenerative changes of a cheesy character, beginning in its centre.

This brief history of the most prominent discoveries and theories regarding the nature of tuberculosis from the time of Hippocrates, to the discoveries of Koch would seem to establish the following propositions:

1. Tuberculosis is an infectious disease.
2. It can be transmitted by inoculation.
3. In certain animals, notably the guinea pig and the rabbit, there exists a predisposition whereby tuberculosis is readily contracted.
4. Tuberculosis occurs almost exclusively in a certain group of persons, who possess a scrofulous diathesis.
5. The anatomical features of tuberculosis is the nodule or tubercle; a growth of definite and specific constitution, which does not differ in its cellular elements from other tissue formations.

It is, therefore, clearly seen that the infectious cause of phthisis remained undiscovered until the 24th day of March, 1882, when Dr. Robert Koch read a paper on the "Etiology of Tubercular Diseases," before the Physiological Society of Berlin, in which he claimed that this infection (hitherto unknown) was a micro-organism ; a "rod-shaped Bacterium," which he called the "Baccillus Tuberculosis." He subjected the tuberculous lungs of a great many men and animals to microscopic examination, and found the nodules infested with this baccillus which he diffenentiated from the surrounding tissue by means of a special dye, or analine staining fluid. It was, he says, "impressive in the highest degree, to find in the center of the tubercle cells the organism which had created it." Transferring directly by inoculation the diseased matter, from diseased animals to healthy ones, he, in every instance, reproduced the disease.

To meet the objection that it was not the bacillus itself, but some virus in which it was imbedded in the diseased organ, that was the real contagium, he cultivated his bacilli artificially for long periods of time, and through many successive generations. With a speck of matter, for example, from a tuberculous lung, he infected a substance prepared, after much trial to himself, with the view of affording nourishment to the bacilli. Here he permitted it to grow, and multiply. From this generation, he took a minute sample, and infected therewith fresh nutritive matter, thus producing another brood. Generation after generation of bacilli were produced in this way. At the end of these cxperiments, which embraced, sometimes, successive cultivations extending over eighteen months, these purified bacilli were introduced by inoculation into the bodies of healthy animals of various kinds. In every instance, such inoculations were followed by the reproduction and spread of the bacilli, and the generation of the original disease. For obvious reasons, these inoculation experiments have been confined to the lower animals.

An interesting case of such inoculation in the human subject is reported in the *New York Medical Record*, for February 14th, of the last year. The subject of this report was a young woman 24 years of age, employed as a cook in the family of a certain Prof. H. She was a strong, healthy girl, who had never had a suspicion of any scrofulous or tuberculous affection, and who gave an unexceptionable family history, free from the slightest taint of tuberculosis. Prof. H. died, in July of last year, of pulmonary phthisis, after an illness of only five or six months duration. Towards the close of his life, the sputum was so loaded with the bacilli of tuberculosis, as to constitute almost a pure culture of these organisms in pus. A few days before her master's death, the cook broke a glass which the master had used to expectorate in, and ran a small sliver into her left hand, inflicting a puncture wound on the palmar surface of the first phalanx of the middle finger. Fourteen days later she presented herself at Professor Studgard's clinic, with what seemed to be a commencing felon. At the end of some weeks, a little nodule, scarcely half so large as a pea, was felt in the subcutaneous connective tissue. During the following week, this remained stationary in size, but became somewhat tender, as was surrounded by an edamatous area. The nodule was now excised, and found to consist of granular matter lying between the sheath of the tendon, and the skin. The wound healed by the first intention. At the beginning of October, the patient again presented herself, complaining of pain on flexing the finger. The subcutaneous connective tissue on the palmar surface of the hand, as well as that of the finger, was swollen, but no distinct nodule or tumor could be discovered. A month later, however, a distinct thickening of the sheath of the tendon was to be felt. Two cubital and two axillary glands were also enlarged. Prof. Studsgard disarticulated the middle finger at the metacarpo-phalangeal joint and dissected away the tendon with its thickened sheath, as far as the middle of the palm. The swollen glands at the elbow and in the axilla were also removed. Examination showed the sheath of the tendon filled with pale granulations. No pus or cheesy matter was to be seen, nor was there any disease of the bones or joints of the amputated finger. The granulations, hardened in alcohol, and stained with picro carmine, when placed under the microscope, were seen to be composed of tubercles, presenting the central caseous degeneration, and containing numerous large and several beautiful giant cells. Tubercles were also found in the enlarged glands. In every section, either of the sheath of the tendon, or of the lymphatic glands, numerous tubercle bacilli were seen, lying either in the giant cells, or at the edges of the microscopical points of the necrobiosis.

Since Dr. Koch first announced his famous discovery, Doctor Formad, of Philadelphia, has repeatedly and emphatically declared that he is able to produce tuberculosis in rabbits, and other animals, by injecting into the abdominal cavity finely powdered inorganic material, such as glass, and ultra-marine blue. His method consisted in first making an incision through the integument over the belly ; then plunging a canula and trocar into the cavity of the abdomen, and finally injecting the sterilized material through the canula with a syringe. He says that about one-third of the animals so operated upon, died during the first week, from septicæmia, and that a certain number of the survivors suffered an acute inflammation, attended with the formation of pus, and its subsequent discharge through the walls of the abdomen.

Experiments of this sort, in order to be trustworthy, should be performed with sufficient care to prevent all other organisms than those intended, from gaining an entrance into the body. This care does not appear to have been taken by Dr. Formad in his inoculations. They were made in his laboratory where these and similar experiments had previously been made upon animals. In such an atmosphere we could certainly expect the presence of many kinds of bacteria—those of tuberculosis included. His operations were not made in such a manner as to exclude the bacilli tuberculosi, if in the atmosphere, or attached to his instruments. A more important objection attaches to the imperfectly closed condition in which he left the wound through the integument. Is it surprising under these circum- that one-third of those acted on died from septicæmia ? A large number had acute inflammation, followed by formation of pus, and its discharge through the walls of the abdomen ; or that others developed tuberculosis ? Dr. Sternberg has repeated the experiments of Dr. Formad with such precautions as would exclude the introduction of tubercle bacilli. He says, "I was particularly desirous that the experiments should be made in a way which would be satisfactory to my friend, Dr. Formad.

I accordingly invited him to assist in making the experiment, and he kindly came from Philadelphia on the the day appointed for the purpose. The powdered glass, used for the experiment, was prepared by Dr. Formad. The amount, used in each case, considerably exceeded a drachm. In all, fifteen rabits were operated upon. These rabits were kept in the country, several miles from Baltimore, in a vacant loft, which had not been previously used for any similar purpose. The inflammation, which resulted from the injection, was usually mild in type, and with two or three exceptions, the animals did will, and remained in good health until they were killed. The experiments were made under the most perfect antiseptic precautions. The substances injected into the abdominal cavities of the animals were thoroughly sterilized, and all necessary precautions used in the manner of their introduction. The animals were killed at various times after being inoculated, some as late as three months. In not a single instance was tubercle, or anything resembling it, found in the tissues of such animals.'' In conclusion, Dr. Sternberg says: ''It is unnecessary to say that this experiment gives no support whatever to the claim that tuberculosis may be induced by injecting into the abdominal cavity of rabbits, finely powdered inorganic particles, or to the view that the tubercle bacillus induces tuberculosis by acting simply as a mechanical irritant.''

It may, therefore, be accepted as an established fact that tuberculosis is an infective disease, induced by the presence of a specific bacillus. In the light of this knowledge, the various theories which have been advanced with regard to the causation of tuberculosis becomes in some respects irrelevent. Clinical experiment would seem to indicate that the tubercle bacillus is not an ordinary bacterium, such as may enter and effect any organism without distinction. It would seem, rather, as if infection occurred only where a definite predisposition exists, or where a considerable quantity of the virus is introduced. Koch finds that the bacilli grow very slowly, and can be bred only between the temperatures of 86o F. and 105o F.; that after inoculation they proceed to develop and multiply only when they reach a spot where they are not subject to much mechanical disturbance or displacement. From this, we may understand how it happens that many persons, though again and again exposed to the invasion of the tubercle bacilli, remain uninfected. It is, moreover, conceivable that individuals, in whose tissues inflammatory changes have occurred, especially those characteristic of scrofula, are those who are most disposed to tuberculous infection.

It is of special interest to note that the bacillus has been found in the sputa of phthisical patients, as the bacilli produce spores within its body which possess resisting powers to the temperature changes and desiccation, not possessed by the bacillus itself; the sputa containing such spores may be a frequent means of conveying the disease.

This special bacillus is found, not only in general miliary tuberculosis, but in caseous pneumonia, and caseous bronchitis, intestinal and glandular tuberculosis of various animals; in the so-called scrofulous hyperplasia of lymphatic glands, etc., and, also, in the pearly disease of cattle.

It would be interesting to know whether the bacillus is constant in the milk of cows so affected. If so, this may eventually prove to be a frequent cause of tuberculosis infection.

When the bacilli of tuberculosis are found in the sputum of a person, it is positive evidence that this person has phthisis. Many cases of tuberculosis can be positively diagonosed by this means, when other methods are insufficient to render the diagnosis positive.

The method of staining the bacilli of tuberculosis, which I have adopted, is the one now employed by Koch.

It is prepared as follows :

| | | | |
|---|---|---|---|
| ℞ | Analine Water (sat. sol), | c. c. | 100 |
| | Alcoholic Solution Fuchsin, | c. c. | 11 |
| | Absolute Alcohol, | c. c. | 10 |
| | Mix. | | |

The analine water is made by thoroughly shaking a few drops of analine oil in distilled water until no more oil will dissolve, and then filtering it through a moistened filter.

Select from the sputum, to be examined, one of the small, white bodies often found in such sputa; if these are not present, then select its firmest portion, and crush a small quantity of this between microscopic cover glasses—rubbing them together until the substance is evenly and thinly spread over the surfaces—they are then to be dried by passing them through the flame of a Bunsen burner, or alcohol lamp. After this, float them, sputum side downward, on the staining fluid, in a shallow vessel, (a watch crystal answers the purpose very well); let them remain at least twelve hours in this stain. They can be stained more quickly, in twenty or thirty minutes, by heating the stain whilst the cover glasses float on its surface, but such specimens lose their color in a short time, and are not fit for permanent preservation. After removal from the staining fluid, immerse the glasses for one second in a solution of nitric acid, one part of acid, two parts of distilled water, then in distilled water, and lastly wash, by passing the glasses to and fro in 60 per cent. alcohol, until all appreciable stain is removed. Lay the specimens aside, protected from the dust, to dry, or they can be dried by passing them a few times through the flame of your alcohol lamp. They are then ready for mounting, which should be done in Canada balsam, softened by the addition of benzole or chloroform, so that it will readily drop from the end of a glass rod. Place a small drop of the balsam on the center of the sputum side of the cover glass, and carefully press it down on your slide so that no air bubbles will be entangled. Under the microscope the bacilli will be seen as delicate red rods, exceedingly small in size.

The bacilli of tuberculosis and those of leprosy are the only ones which will retain this dye stain after immersion in nitric acid, and subsequent washing in alcohol.

The histological microscopic stand, Abbe's condenser, modified, and the one-sixth object glass, as manufactured by J. Grunow, of New York City, comprises the instrument which I use in examining the bacilli for purpose of diagnosis ; and I find this power, with the light given by the Abbe's condenser, amply sufficient for this purpose.

